# Clinical and radiographic comparison of a single LP-PRP injection, a single hyaluronic acid injection and daily NSAID administration with a 52-week follow-up: a randomized controlled trial

**DOI:** 10.1186/s10195-018-0501-3

**Published:** 2018-08-20

**Authors:** David Buendía-López, Manuel Medina-Quirós, Miguel Ángel Fernández-Villacañas Marín

**Affiliations:** 1 Hospital Caravaca de la Cruz, Avenida Miguel Espinosa, 1, Caravaca de la Cruz, 30400 Murcia, Spain; 2 Hospital Virgen de la Arrixaca, Carretera Madrid-Cartagena, El Palmar, 30120 Murcia, Spain; 3 Human Anatomy Department and Psychobiology, Murcia University, Campus de Espinardo, 30100 Murcia, Spain

**Keywords:** Knee osteoarthritis, Platelet-rich plasma, Hyaluronic acid, Intra-articular injection, Cartilage injury

## Abstract

**Background:**

Knee osteoarthritis (OA) is a disease with a high prevalence in the adult population. Nonsteroidal anti-inflammatory drugs (NSAID) or intra-articular injections [hyaluronic acid (HA) or platelet-rich plasma (PRP)] can provide clinical benefit. Magnetic resonance imaging (MRI) has proven to be useful for the evaluation of cartilage volume and thickness in knee osteoarthritis. The purpose of this study was to evaluate the benefit provided by PRP injection in comparison with hyaluronic acid and NSAID in knee OA patients and to compare the radiographic evolution at the 52-week follow-up.

**Methods:**

One hundred and six patients were enrolled and randomized according to the Spanish Rheumatology Society knee osteoarthritis diagnosis criteria. Ninety-eight patients completed the study (33 received NSAID treatment, 32 a single hyaluronic acid injection and 33 a single PRP injection). Patients were prospectively evaluated at baseline, 26 and 52 weeks using the Western Ontario McMaster Universities osteoarthritis index (WOMAC) and the visual analogue scale (VAS), and at baseline and 52 weeks with X-ray and MRI.

**Results:**

A 20% decrease in WOMAC pain and increase in physical function was found in 30 and 24%, respectively, of those patients who received PRP treatment, at the 52-week follow-up. WOMAC pain and VAS improved in the hyaluronic acid and NSAID groups. However, better results were obtained in the PRP group compared to hyaluronic acid and NSAIDs (*P* < 0.05). No differences in Kellgren–Lawrence or cartilage thickness progression were found.

**Conclusions:**

Leukocyte-poor platelet-rich plasma (LP-PRP) injections are better in terms of clinical improvement with respect to HA injections or oral NSAID treatment in knee osteoarthritis patients at the 52-week follow-up. Moreover, a single LP-PRP injection is effective. However, LP-PRP has no influence on cartilage progression.

**Level of evidence:**

Level II.

## Introduction

Every day, orthopaedic surgeons face the problem of osteoarthritis (OA), with a prevalence increasing day after day [1]. Moreover, this disease has a devastating impact on a patient’s quality of life and it has become the most common degenerative joint disorder in the elderly [2, 3].

From a structural point of view, even though osteoarthritis affects the whole joint, cartilage degeneration characterises this disease [4]. In this sense, despite the number of different treatments available, there are no medical treatments that can change the natural course of the disease and prevent cartilage from degenerating. Several studies have looked at the effectiveness of oral substances such as non-steroidal anti-inflammatory drugs (NSAID), analgesics, and symptomatic slow action drugs for osteoarthritis (SYSADOA) such as hyaluronic acid, keratin or chondroitin sulphate [5, 6]. Intra-articular steroid injections have demonstrated short-term effects on knee pain and disability [7]. Moreover, intra-articular hyaluronic acid (HA) injections represent an effective and safe method, without increased risk of adverse events, in the treatment of pain and joint dysfunction in osteoarthritis of the knee [8].

One of those treatments, platelet-rich plasma (PRP) is described as an autologous blood product with an increased concentration of platelets. Several studies have shown the use of this biological therapy as clinically effective in osteoarthritis of the knee [9]. However, no therapeutic option is considered ideal for OA [10]. The use of PRP therapy is associated with a reduction in tissue inflammation and represents an option for cartilage injuries in osteoarthritis [11]. There is no clarity in reference to the number and frequency of PRP injections. Despite the clinical results, many questions remain unanswered regarding the efficacy of PRP [12]. For example, the presence of leukocytes in the PRP preparation was believed to cause more adverse events (pain and swelling) [13]. However, a recent meta-analysis carried out by Görmely [10] has demonstrated that there is no difference in adverse events when comparing PRP preparations with different leukocyte concentrations.

With respect to radiography, at present, joint space narrowing and Kellgren–Lawrence progression from serial radiographs are the accepted structural endpoints used in clinical trials [14]. Moreover, quantitative magnetic resonance imaging (MRI) has proven to be useful for the evaluation of cartilage volume and thickness in knee osteoarthritis [15].

To our knowledge, there is no prospective randomized study in the literature comparing the clinical effectiveness of a single PRP injection, a single hyaluronic acid injection and the presence of an intra-articular control group in patients affected by osteoarthritis of the knee, with the use of X-ray (Kellgren–Lawrence progression) and MRI (responsiveness of quantitative cartilage measures) over a 52-week follow-up.

The purpose of this study was to evaluate the safety and clinical efficacy of a single LP-PRP (leukocyte-poor platelet-rich plasma) injection in the management of osteoarthritis of the knee, compared with a single HA injection and the use of NSAID, and to compare the Kellgren–Lawrence progression and responsiveness of quantitative cartilage measures in MRI. We considered a 20% reduction in the WOMAC pain subscale from baseline as the primary outcome and a 20% reduction of WOMAC stiffness, physical function subscales, VAS, and X-ray and MRI progression as secondary outcomes. We hypothesized that a single LP-PRP injection would be more effective in reducing pain and improving joint function than a single HA injection or the use of oral NSAID from baseline to week 52. We also hypothesized that a single LP-PRP injection would improve the Kellgren–Lawrence progression and the responsiveness of quantitative cartilage measures in MRI compared with a single HA injection and the use of oral NSAID from baseline to week 52.

## Materials and methods

This study was designed as a prospective and randomized trial with 3 groups and 3 treatment methods (the PRP group, receiving 1 PRP injection; the HA group, receiving 1 HA injection and the non-intra-articular group, receiving a daily NSAID dose).

### Patient selection

All patients provided written informed consent before entry to the study. From April 2013 to November 2013, 124 patients were screened and finally 106 were randomized for this study, being the starting point of the groups of treatment from December 2013 through May 2014. Patients were followed up at 6 and 12 months, until May 2015. Eligibility criteria were: symptomatic knee osteoarthritis as defined by the Spanish Society of Rheumatology (based on the Altman osteoarthritis for the knee criteria [16], combining both clinical and radiographic criteria with a 91% sensitivity and 86% specificity) and Kellgren–Lawrence grade of 1 or 2. Patients were excluded if they had a varus deformity of > 4.2° (moderate varus) [17] or a valgus deformity, recent trauma, inflammatory arthritis, history of gastrointestinal or cardiovascular disease, concomitant medications of potent analgesics, corticosteroid, NSAID, anticoagulant or antiplatelet therapy within 12 months of study enrolment; previous surgery to the limb or spine; previous injection to study joint or any active local or systemic infection; systemic disorders with restrictions for the use of NSAID (diabetes) or potential effect on the knee (rheumatic, metabolic, musculoskeletal or neuropathic disorders). Patients were only included in the study if they met all the inclusion/exclusion criteria (106 of 124 because 18 were excluded). In the patients with bilateral symptoms, only the side with significant symptoms was taken into account.

**Fig. 1:**
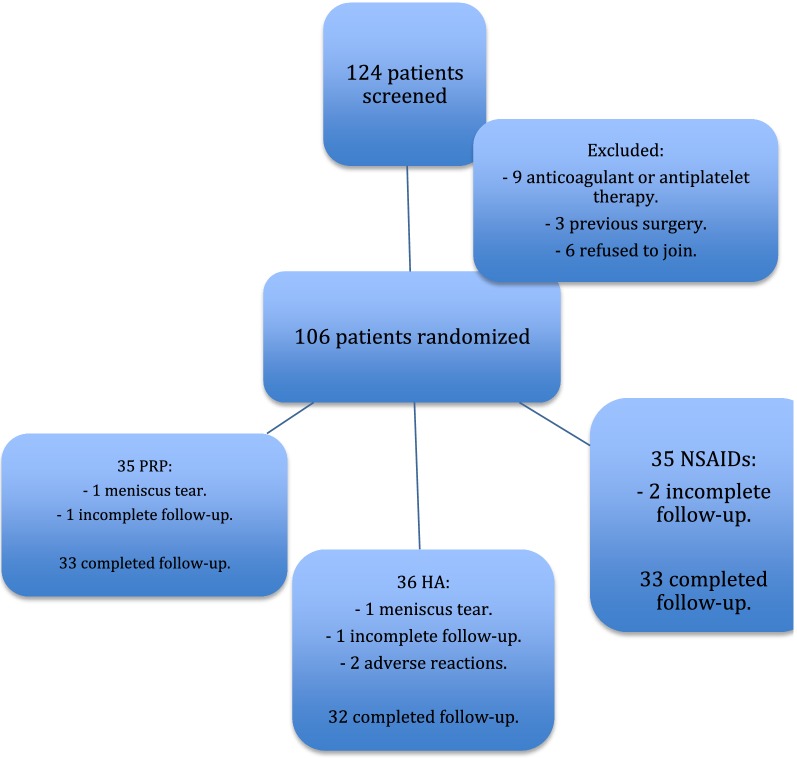
Patients screened, randomized and exclude

To sum up, a total of 124 patients were initially screened, and 18 were excluded because they did not meet all the inclusion/exclusion criteria; thus 106 patients underwent randomization and treatment, and 98 patients completed the follow-up. Figure 1 shows the number of patients screened, randomized and excluded.

The mean age was 56.82 years (range 50–63 years), the mean body mass index was 25.1 (range 23.8–26.1). Table 1 shows the number of subjects in every group, their demographic data, Kellgren–Lawrence grade, WOMAC subscales and VAS at baseline.

**Table 1:** Patient characteristics

	PRP	HA	NSAID
Age	56.15 ± 3.001	56.63 ± 2.9	57.42 ± 3.1
BMI	24.9 ± 0.32	24.9 ± 0.41	25.2 ± 0.48
Gender (M/F)	16/17	15/17	16/17
Kellgren–Lawrence (1–2)	18/15	18/14	17/16
WOMAC score (baseline)	42.57 ± 7.3	42.62 ± 7.3	42.66 ± 7.8
Pain	6.09 ± 1.4	6.03 ± 1.2	6.12 ± 1.2
Stiffness	4.12 ± 0.7	4.06 ± 1.2	4.06 ± 0.8
Physical function	32.36 ± 5.9	32.53 ± 7.1	32.48 ± 6.8
VAS	6.15 ± 1.1	6.06 ± 0.9	6.15 ± 1.2
Number of patients	33	32	33

*M* male, *F* female

### Interventions

The clinical examination of the volunteers included the Western Ontario and McMaster Universities osteoarthritis index (WOMAC) questionnaire and the visual analogue scale (VAS). The radiographic examination included radiographs of the lower limbs under loading of the affected knee in the Rosenberg X-ray projection (a posteroanterior weight-bearing in 45° of flexion projection) and a lateral (full-extension) projection. We also included magnetic resonance imaging (MRI) to assess chondral degeneration. Mean cartilage thickness normalized to the total area of subchondral bone was obtained for a total of 16 locations in the femur and 24 in the tibia, distributed in five tibial subregions (central, external, internal, anterior and posterior) and three femoral subregions (central, external and internal). Magnetic resonance imaging data were acquired on Siemens MAGNETOM Essenza 1.5 T, extremity coil. The imaging protocol included sagittal spin-echo proton density- and T2-weighted images [repetition time (TR), 2200 ms; time to echo (TE) 20/80 ms] with a slice thickness of 3 mm, a 1-mm inter-slice gap, 1 excitation, a field of view (FOV) of 12 cm, and a matrix of 256 × 192 pixels; and coronal and axial spin echo fat-suppressed proton density- and T2-weighted images (TR 2200 ms; TE 20/80) with a slice thickness of 3 mm, and a 1-mm inter-slice gap. We used a positioning device to ensure uniformity among patients. Patients remained in the supine position with a fully extended knee and the foot perpendicular to the MRI table. Each cartilage measure (cartilage defects, full-thickness cartilage) and X-ray data were read by one trained musculoskeletal radiologist, blinded to the groups of treatment, using the software OsiriX (6.0.2 for Mac). Bone marrow lesion (BML) was defined as an area of increased intensity in subchondral bone in the distal femur and proximal tibia.

The clinical examination was repeated at 6 and 12 months, and the radiographic examination was repeated at 12 months after treatment. A total of 98 of the 106 participants were prospectively evaluated at 12-month follow-ups. Two suffered from a meniscus tear, 2 had arthritis post-injection which required the use of NSAID and 4 were lost to follow-up.

### Randomization

During the patient visits, the treatment was assigned by a simple randomization after signing the informed consent form. Each patient was identified by a numerical code and treatment was assigned using free randomization software (http://www.randomizer.org).

### Treatment

The PRP group received a 5-ml PRP injection. Each patient had 60 ml of peripheral blood extracted by venipuncture of the antecubital vein. A double centrifugation process was carried out. The first spin step was 1050 rpm for 15 min and for the second spin step, an acceleration of 2000 rpm for 10 min was applied. A total 5 ml of an LP-PRP preparation was obtained, being activated by 1 ml of calcium chloride. Five patients of the PRP group were selected by lot to get a double preparation in order to find out the platelet concentration. The platelet concentration was 1,095,000 ± 23,200/mm^3^, which was 3.87 times greater than the baseline concentration.

In the HA group, patients were treated with a single high molecular weight preparation (60 mg/2 ml, Durolane©). The control group received a daily NSAID dose (60 mg etoricoxib, Acoxxel©) for 52 weeks. We co-prescribed a proton pump inhibitor (20 mg omeprazol a day). The patients were evaluated before treatment and at 6- and 12-month follow-ups.

### Outcome measures

The primary efficacy outcomes were defined as the percentage of patients having a 20% decrease for the WOMAC pain subscale from baseline. The secondary efficacy outcomes included a 20% decrease for the WOMAC stiffness, physical function, VAS, and X-ray and MRI progression. The nature, duration and severity of any adverse event related to the study medication was assessed.

### Sample size and statistical analysis

We used GPower software for the sample size estimation. We estimated a sample size of 28 patients per group to provide at least 80% power to detect differences in the WOMAC pain scale superior to 1 for PRP injection versus HA, at 5% level of significance, taking into consideration 10% possible losses. The minimal clinically relevant difference was a change of 20% for VAS and WOMAC subscales. Quantitative variables (age, BMI, VAS, WOMAC subscales and cartilage thickness) were determined by the mean, standard deviation and range. For qualitative variables (gender, Kellgren–Lawrence grades, and treatment group) a frequencies analysis was conducted. A descriptive analysis of the sample was performed, taking into consideration the demographic, clinical and radiographic variables. The quantitative data were compared by analysis of variance (ANOVA) followed by Tukey’s HSD test. The qualitative data were analysed using Pearson’s chi-square test. For all outcomes, a nominal *P* value of less than 0.5 was considered to indicate statistical significance. All the analyses were conducted with IBM SPSS software, v.21.0 for Windows.

## Results

At baseline, we used the analysis of variance (ANOVA) to determine whether there were any statistically significant differences between the means of quantitative variables (age, BMI, WOMAC subscales, VAS and cartilage thickness) in the three groups of treatment. No statistically significant differences were found. The follow-up time was 26 weeks (range 25 weeks 6 days, 26 weeks 1 day) and 52 weeks (range 51 weeks 6 days, 52 weeks 2 days).

### Clinical outcomes

#### Twenty-six weeks

Results of primary and secondary outcome measures at 26 weeks for the entire population and all WOMAC and VAS scores are summarized in Table 2.

**Table 2:** Outcomes at 26 weeks

	PRP	HA	NSAID	*P*
Patients	33	32	33	
Responders [no. (%)]				
20% decrease WOMAC pain	16 (48)	7 (21)	5 (15)	< 0.001
20% decrease WOMAC stiffness	15 (45)	5 (15)	4 (12)	< 0.002
20% decrease WOMAC physical function	15 (45)	5 (15)	4 (12)	< 0.05
20% decrease VAS	16 (48)	8 (25)	6 (18)	< 0.021
Change from baseline				
WOMAC pain				
% change from baseline	− 22.38	− 14.5	− 5.9	< 0.001
End of follow-up	4.72 ± 0.87	5.15 ± 0.84	5.75 ± 0.43	< 0.005
WOMAC stiffness				
% change from baseline	− 18.3	− 0.5	2.9	< 0.05
End of follow-up	3.36 ± 0.5	3.56 ± 0.5	4.18 ± 0.39	< 0.001
WOMAC physical function				
% change from baseline	− 21.1	− 12	0.6	< 0.001
End of follow-up	± 0.6	28.62 ± 0.9	32.69 ± 0.8	< 0.001
WOMAC total				
% change from baseline	− 21.06	− 12,39	− 0.06	< 0.03
End of follow-up	33.6 ± 1.2	37.34 ± 1.2	42.63 ± 1.02	< 0.002
VAS				
% change from baseline	− 20.2	− 13.92	− 5.4	< 0.001
End of follow-up	4.9 ± 0.52	5.21 ± 0.6	5.81 ± 0.39	< 0.001

A primary response was defined as the percentage of patients having a 20% decrease in the summed score for the WOMAC pain from baseline to week 26. Quantitative variables are expressed as mean standard deviation. *P* < 0.05 is considered statistically significant

Regarding the primary outcome measure (the percentage of patients having a 20% decrease for the WOMAC pain), the results were significantly different in the three treatment groups.

Comparing the PRP and HA groups and regarding the primary outcome, the rate of response to PRP was 27 percentage points (95% confidence interval [CI] 21–29; *P* < 0.05), higher than the rate of response to HA for the WOMAC pain. Regarding the secondary outcomes (the percentage of patients having a 20% decrease for the WOMAC stiffness and physical function, and VAS) the rate of response to PRP was 30 percentage points for WOMAC stiffness (95% CI 27–32; *P* < 0.05), 30 percentage points for WOMAC physical function (95% CI 26–32; *P* < 0.05) and 23 percentage points for VAS (95% CI 19–25; *P* < 0.05) higher than the rate of response to HA.

Comparing PRP and NSAID and regarding the primary outcome measure (the percentage of patients having a 20% decrease for the WOMAC pain), the rate of response to PRP was 30 percentage points (95% confidence interval [CI] 26–32; *P* < 0.05) higher than the rate of response to NSAID. Regarding the secondary outcomes, the rate of response to PRP was 33 percentage points for WOMAC stiffness (95% CI 28–34; *P* < 0.05), 33 percentage points for WOMAC physical function (95% CI 28–35; *P* < 0.05) and 30 percentage points for VAS (95% CI 27–32; *P* < 0.05) higher than the rate of response to NSAID.

Comparing HA and NSAID, the rate of response to HA for the WOMAC subscales and VAS was slightly superior although there was no statistically significant difference.

Table 2 shows the response in each group of treatment for all the scores.

#### Fifty-two weeks

Results of primary and secondary outcome measures at 52 weeks for the entire population and all WOMAC and VAS scores are summarized in Table 3. The results obtained at 52 weeks followed the same trend as those at 26 weeks.

**Table 3:** Outcomes at 52 weeks

	PRP	HA	NSAID	*P*
Patients	33	32	33	
Responders [no. (%)]				
20% decrease WOMAC pain	10 (30)	0	0	< 0.001
20% decrease WOMAC stiffness	9 (27)	0	0	< 0.001
20% decrease WOMAC physical function	8 (24)	0	0	< 0.05
20% decrease VAS	5 (15)	0	2(6)	< 0.001
Change from baseline				
WOMAC pain				
% change from baseline	− 20.39	− 1.03	− 6.4	< 0.03
End of follow-up	4.84 ± 0.7	5.96 ± 0.4	5.72 ± 0.45	< 0.001
WOMAC stiffness				
% change from baseline	− 16.1	− 0.7	4.9	< 0.001
End of follow-up	3.45 ± 0.5	4.03 ± 0.3	4.27 ± 0.45	< 0.002
WOMAC physical function				
% change from baseline	− 19	0.3	0.9	< 0.001
End of follow-up	26.21 ± 0.8	32.65 ± 0.7	32.78 ± 0.73	< 0.05
WOMAC total				
% change from baseline	− 18.9	0.07	0.2	< 0.001
End of follow-up	34.51 ± 1.2	42.65 ± 0.9	42.78 ± 1.02	< 0.02
VAS				
% change from baseline	− 18.2	3	− 6.4	< 0.001
End of follow-up	5.03 ± 1.7	6.25 ± 0.4	5.75 ± 0.43	< 0.001

A primary response was defined as the percentage of patients having a 20% decrease in the summed score for the WOMAC pain. Quantitative variables are expressed as mean standard deviation. *P* < 0.05 is considered statistically significant

Comparing the PRP and HA groups and regarding the primary outcome (a 20% decrease for the WOMAC pain) the rate of response to PRP was 30 percentage points (95% confidence interval [CI] 27–32; *P* < 0.05) higher than the rate of response to HA for the WOMAC pain subscale. Regarding the secondary outcomes the rate of response to PRP was 27 percentage points for WOMAC stiffness (95% CI 25–28; *P* < 0.05), 24 percentage points for WOMAC physical function (95% CI 23–26; *P* < 0.05) and 15 percentage points for VAS (95% CI 14–17; *P* < 0.05) higher than the rate of response to HA.

Comparing PRP and NSAID groups, and regarding the primary outcome, the rate of response to PRP was 30 percentage points for WOMAC pain (95% confidence interval [CI] 28–32; *P* < 0.05) higher than the rate of response to NSAID. Regarding the secondary outcomes, the rate of response to PRP was 27 percentage points for WOMAC stiffness (95% CI 25–28; *P* < 0.05) and 24 percentage points for WOMAC physical function (95% CI 23–26; *P* < 0.05) higher than the rate of response to NSAID. Regarding the VAS, no statistically significant difference was found.

Comparing HA and NSAID at 52 weeks, the rate of response to HA and NSAID for the WOMAC subscales and VAS did now show statistically significant difference.

Table 3 summarizes the response in each group.

### Radiographic outcomes

The overall progression in the Kellgren–Lawrence score for the whole group was 17% from grade 1 to grade 2, from baseline to week 52. In our study there was no worsening from grade 2 nor 2 or more grades from grade 1. We could not see reduction of Kellgren–Lawrence in any patient. Comparing the PRP, HA and NSAID groups, no statistically significant difference was found.

Analysis of cartilage thickness (Table 4) showed reduction in all tibial and femoral subregions in the three groups, from baseline to week 52. Comparing the PRP, HA and NSAID groups, no statistically significant difference was found in reference to the cartilage thickness reduction. In our study, we did not observe cartilage thickening in any subregion, femoral or tibial.

**Table 4:** Cartilage thickness at baseline and 52 weeks

Cartilage thickness (mm)	Baseline	52 weeks
Femoral subregions		
Central	1.73 ± 0.4	1.68 ± 0.32
External	1.31 ± 0.31	1.25 ± 0.27
Internal	1.83 ± 0.26	1.78 ± 0.42
Tibial subregions		
Anterior	1.42 ± 0.26	1.38 ± 0.3
Posterior	1.28 ± 0.2	1.22 ± 0.27
Central	1.82 ± 0.4	1.78 ± 0.35
External	1.51 ± 0.31	1.46 ± 0.28
Internal	1.68 ± 0.25	1.62 ± 0.28

Cartilage thickness normalized to the total area of subchondral bone (mean ± SD)

### Adverse effects

Over the 52-week follow-up, only 2 adverse events were reported during the study, both in the HA group. These events were related to pain and swelling, related to HA infiltration, in the immediate period after the infiltration (2 weeks). Both patients required the use of NSAID for over a week and were withdrawn from the study. No other adverse events were related to the use of PRP, HA or NSAID.

## Discussion

This prospective and randomized study reported the effect of LP-PRP, HA and the use of oral NSAID on osteoarthritis of the knee. The most important finding of this study was that a single course of LP-PRP resulted in clinical efficacy (a reduction of pain and an improvement in physical function) in patients with early osteoarthritis at the 52-week follow-up. Another important result of this study was that a single dose of LP-PRP had a superior clinical effect over a single dose of HA and the use of oral NSAID. In this sense, the response to LP-PRP in all the clinical scores (pain, stiffness and physical function) was better than the response to HA and NSAID.

There are few prospective studies that have evaluated the effectiveness of PRP and the superiority of PRP over HA in the treatment of osteoarthritis [18–22]. Those studies showed that PRP treatment obtained better clinical results than HA. However, one important limitation of those studies was that they did not include a control group. To try to address these limitations, we included a non-intra-articular treatment (NSAID versus intra-articular injections).

As suggested in previous studies [13, 23], the presence of leukocytes could generate a pro-inflammatory environment; more swelling and pain reactions have been reported when using leukocyte-rich PRP (LR-PRP). That is the reason why we used an LP-PRP preparation in this study.

With respect to the clinical effect of the concentration of platelets in the PRP preparation, a previous study by Filardo et al. [13] showed that different concentrations of platelets produced comparable clinical results. Therefore, a higher amount of platelets would not produce a different clinical outcome. In our study, on average, the LP-PRP injection had 3.87 times as many platelets as did whole blood.

Concerning the clinical effect of LP or LR-PRP, in a meta-analysis carried out by Riboh [24], it was concluded that LP-PRP preparations improved functional outcome scores compared to hyaluronic acid and placebo in patients affected by knee osteoarthritis. With regard to the use of LP- or LR-PRP, a recent review [25] showed that there is limited evidence when comparing the clinical outcomes of LR- versus LP-PRP. We used PRP rich in platelets and poor in leukocytes, in agreement with previous studies, to obtain clinical efficacy and reduce adverse events such as pain or swelling. However, the ideal leukocyte concentration is under debate and further randomized trials are needed to compare the clinical efficacy of LR- and LP-PRP.

Due to the fact that previous studies included different grades of knee osteoarthritis (from I to IV according to Kellgren–Lawrence), the response to treatment was highly variable. However, Görmeli [10] concluded that PRP injections are useful in achieving better clinical results in early osteoarthritis, compared to hyaluronic acid. We decided to include only grades I and II in order to create a homogeneous study group. There was no statistically significant difference in the clinical response between both grades and with reference to any treatment.

Regarding radiographic progression, previous studies [26–28] showed that treatments such as PRP or HA have no significant influence on cartilage condition evaluated by MRI. In our study, no statistically significant difference was found in the Kellgren–Lawrence progression or in the responsiveness of quantitative cartilage measures in MRI. Moreover, PRP, HA or NSAID could not reduce the Kellgren–Lawrence scores or achieve any cartilage thickening in any femoral or tibial subregion.

This randomized clinical trial reinforces the idea that PRP is secure and effective in the treatment of knee ostearthritis patients, superior to treatment with hyaluronic acid or NSAID, and with positive clinical effects lasting for 52 weeks.

The limitations of this study include the lack of a placebo group and being a single-blind study. It is true that the different treatments (injection versus oral treatment) makes it almost impossible to blind the patients. However, the evaluation of the patients was performed in a blinded way.

LP-PRP injections are better in terms of clinical improvement with respect to HA injections or oral NSAID treatment in knee osteoarthritis patients at the 52-week follow-up. Moreover, a single LP-PRP injection is effective. However, LP-PRP has no influence on cartilage progression.
